# Machine learning estimates on the impacts of detection times on wildfire suppression costs

**DOI:** 10.1371/journal.pone.0313200

**Published:** 2024-11-20

**Authors:** Michael Shucheng Huang, Bruno Wichmann

**Affiliations:** Department of Resource Economics and Environmental Sociology, University of Alberta, Edmonton, Alberta, Canada; University of Vermont, UNITED STATES OF AMERICA

## Abstract

As climate warming exacerbates wildfire risks, prompt wildfire detection is an essential step in designing an efficient suppression strategy, monitoring wildfire behavior and, when necessary, issuing evacuation orders. In this context, there is increasing demand for estimates of returns on wildfire investments and their potential for cost savings. Using fire-level data from Western Canada during 2015–2020, the paper associates variation in wildfire reporting delays with variation in suppression costs. We use machine learning and orthogonalization methods to isolate the impact of reporting delays from nonlinear impacts of the fire environment. We find that reporting delays account for only three percent of total suppression costs. Efforts to improve detection and reduce wildfire reporting delays by one hour lead to a modest 0.25% reduction in suppression costs. These results suggest that investments in detection systems that reduce wildfire reporting delays are not justified on suppression costs savings alone.

## 1. Introduction

Wildfire suppression is a priority for areas in which forests and human communities intersect. In the western Canadian province of Alberta, a forested area spanning 39 million hectares, or the size of Germany, is enjoyed by over one million individuals as home, workplace and places of recreation [1]. While wildfire is an integral component in the boreal forest ecosystem [2], out-of-control fires can have high socioeconomic costs. They force emergency evacuations of people [3, 4] as they threaten the destruction of both human communities and wildlife habitat [5]. Wildfires negatively impact air quality [6], and deteriorate respiratory health more than fine particles from other sources of air pollution [7]. Economic effects are also significant as wildfires can cause an array of direct and indirect losses [8], decrease property values [9], earnings and employment [10].

**Fig 1:**
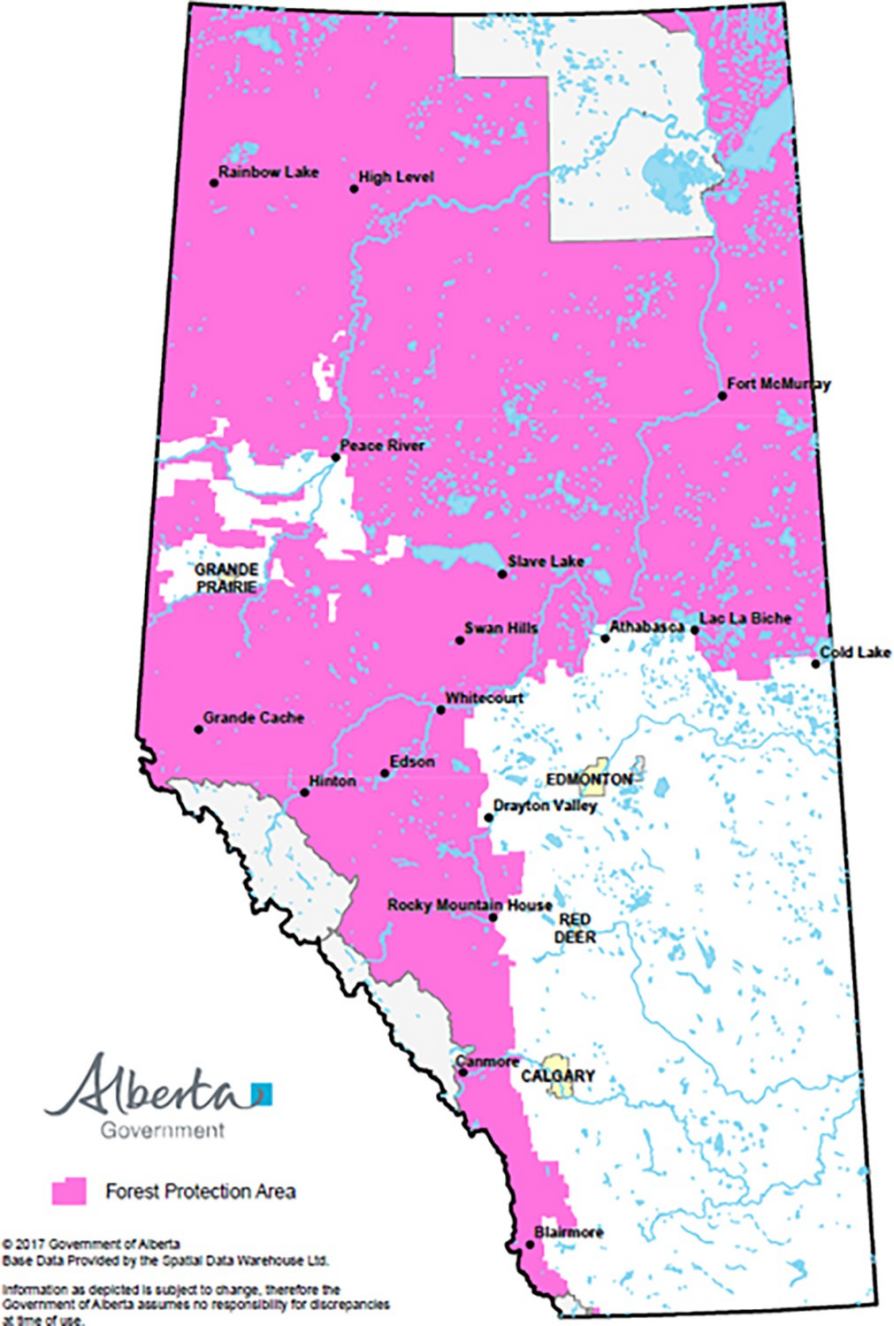
Alberta’s forest protection area. *Source*: Government of Alberta (available online at https://open.alberta.ca/publications/forest-protection-area-map).

Towards protecting communities, industries and natural habitats, the Province of Alberta maintains a policy of total wildfire suppression in the Forest Protection Area (Fig 1). The provincial agency, Alberta Wildfire, is tasked with carrying out suppression programs. The programs are designed with the double objective of safeguarding human and environmental assets, while minimizing operational costs as stewards of public funds.

In the recent 20 years, as suppression costs rise with increasingly severe fire seasons in Canada, there has been growing awareness on the impacts and costs of wildfires [11, 12]. In Alberta, devastating fire seasons in 2016, 2019, and 2023 serve as reminders of wildfires’ economic and social disruptions. In 2016, the damage caused by the 600 thousand hectare Fort McMurray fire, along suppression expenditures, cost of community relocation, and indirect impacts on the environment, totaled an estimated $10.9 billion. Alberta Wildfire expensed over half a billion in suppression operations; additionally, with $3.6 billion in insured property damage, the fire was the costliest insured natural disaster in Canadian history [13–15]. In 2019, nearly a thousand individual wildfires spread across a cumulative 880 thousand hectares, and the expenditures for their suppression totaled $570 million [1]. Finally, the 2023 fire season is responsible for almost two million hectares of area burned, which amounts to almost twice as much the total area burned from the five previous seasons [16]. After incurring $851 million on fire suppression in 2023 [17], the government opted to designate $2 billion in contingency funds for fire and other natural disasters in 2024 [18].

Future prognostics for the threat of wildfires are not encouraging. Across North America, climate change continues to create extreme fire weather conditions [19–23]. Moreover, governments around the world are increasingly challenged to restrain expenditures to balance the public purse. Consequently, wildfire management agencies such as Alberta Wildfire are embattled in facing more challenging wildfires, while also dealing with increased public scrutiny [24] and uncertain budgets [25].

Towards reducing expenditures on wildfire suppression, management agencies are prompted to consider the role of pre-suppression strategies such as early detection mechanisms. Alberta Wildfire recognizes the importance of early detection; it is one of the last Canadian agencies to keep a large network of manned lookout towers, and also engages regularly with the general public to raise awareness on the importance of public reporting of out-of-control wildfires. Through a coordination of manned and unmanned detection tools, including using remote cameras and unmanned aircraft systems (UAS), the agency strives to reduce the delay between a wildfire’s ignition and the time at which the fire is first reported, which we term “reporting delay”.

Wildfire detection is central to management and response. The recent and tragic wildfires in Maui, Hawaii, has brought the issue of timely wildfire detection and community alerts to the forefront of the wildfire management debate [26]. Nevertheless, given limited resources, investments into detection systems can crowd out investments into fire prevention and forest management [27]. As a result, it is important to understand how early detection translates into costs savings and other socioeconomic benefits.

In this paper, we estimate the impact of reporting delays on the cost of suppression, controlling for the fire environment. As fire expenses can be very nonlinear on many important characteristics of the fire environment (e.g. weather), we employ nonparametric empirical models where we make no assumptions about the shape of the influence of the fire environment variables on suppression costs. We estimate marginal effects of reporting delays using a Double/Debiased Machine Learning (DML) algorithm [28]. As we further discuss below, the algorithm allows us to maintain the causal interpretation of the impact of reporting delays on fire suppression costs while utilizing flexible Random Forests algorithms to fit nonparametric fire environment functions. Our models are estimated using fire-level data from 2015 to 2020.

The remainder of the paper is organized as follows: Section 2 summarizes Alberta’s wildfire detection framework. Section 3 describes the data and presents summary statistics. The empirical model and estimation is discussed in section 4. Section 5 presents the results, and section 6 offers concluding remarks, including a discussion on policy implications.

## 2. Wildfire detection in Alberta, Canada

Alberta Wildfire has a mandate in protecting, in descending order of priority, human lives, communities, watersheds and sensitive soils, natural resources, and infrastructure. Towards fulfilling this mandate, the Province of Alberta has adopted strategies for both wildfire prevention and mitigation, as well as for active suppression (more details on mandate priorities and protocol found at: https://www.alberta.ca/how-we-fight-wildfires). Year-round prevention efforts includes public education campaigns on safe burning procedures and permitting, while mitigation includes coordinated fuel reduction activities [29], as well as the Province’s provision of funding to communities to reduce their wildfire risk through taking part in the FireSmart program (https://www.alberta.ca/firesmart). In addition, regional fire bans are implemented during periods of high fire risk (https://www.alberta.ca/fire-bans), further reducing the likelihood of human-caused fires. In our dataset, we find that 56% of fires are linked with human activities such as littering, poorly controlled waste burning, and off-highway vehicle driving.

**Fig 2:**
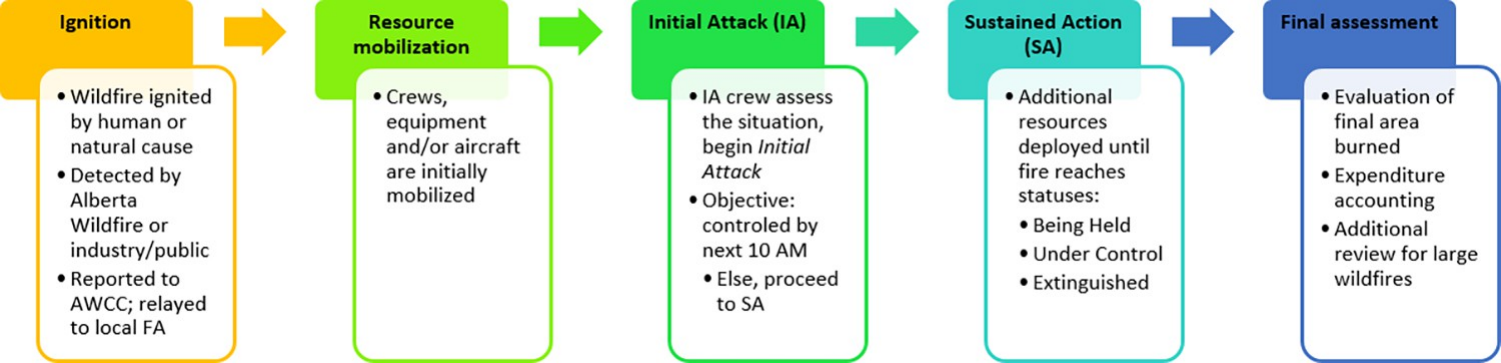
Flowchart of Alberta Wildfire’s response protocol.

Despite the best efforts of Alberta Wildfire and local communities to reduce wildfire risk, ignitions and wildfire spread continues to occur every fire season. When this occurs, Alberta Wildfire initiates a response protocol, as summarized in Fig 2. Response begins as soon as a wildfire is detected in the Forest Protection Area, via the agency’s detection system or as notified by the public. Having received a notification, the Alberta Wildfire Coordination Centre relays the incident to its respective Forest Area region. Resources are then mobilized to the fire for assessment and suppression action. Suppression is terminated when a lead Incident Commander declares the fire to be extinguished, and crews are demobilized after completing a final assessment of the burn area.

Within this strategic framework, the preliminary stages of detection and reporting are critical, because the early and precise detection of wildfires allows decision makers the necessary time and information to implement an appropriate response. Alberta Wildfire classifies fires in five categories, or classes, based on fire size: A: <0.1 ha; B: 0.1–4 ha; C: 4–40 ha; D: 40–200 ha; E: >200 ha. Fire size is continuously monitored. Upon reaching a particular “size class”, a fire will receive certain minimum levels of suppression resources (e.g. crew, equipment and/or aircraft deployed to the fire), as mandated by Alberta Wildfire protocol.

Detection depends largely on regular patrols by Alberta Wildfire crews, as well as on public reporting. In addition, Alberta is one of the last jurisdictions in Canada to keep an extensive network of manned lookout towers. Alberta Wildfire maintains that the towers continue to be critical for precise wildfire detection in a populous wildland-urban interface that covers much of the Forest Protection Area [30].

In addition to traditional detection infrastructure, Alberta Wildfire continues to trial new technologies in order to enhance current detection strategies. In 2021, Alberta Wildfire had invested over $4.3 million in piloting the use of tools such as cameras and unmanned area vehicles, or UAS [30, 31]. This technological renewal sought to improve detection capacity, as well as to help the agency in its resilience to budget shocks that have impacted staffing in recent fire seasons [32]. Infrared imaging via UAS is a highly promising emergent technology, both as an effective wildfire detection tool, and in providing real-time data on fire behavior in areas that are geographically remote or too dangerous for wildland firefighters to access in-person [33–35]. Most recently in 2023, Alberta Wildfire has, for the first time, granted permission for a contractor to fly UAS beyond visual line of sight [36].

## 3. Data

Our data was primarily sourced from Alberta Wildfire’s internal data on operations and expenditure from April 1, 2015 to December 31, 2020. **Table 1** shows the distribution of fires, by size class and year.

**Table 1:** Distribution of wildfires by size class, calendar years 2015 to 2020.

Calendar Year	Size class					Total
	A	B	C	D	E	
	(<0.1 ha)	(0.1–4 ha)	(4-40ha)	(40–200 ha)	(>200ha)	
2015†	1089	514	107	44	64	1818
2016	930	414	62	19	11	1436
2017	852	296	52	24	20	1244
2018	824	349	87	18	21	1299
2019	635	272	61	16	21	1005
2020	574	129	16	1	3	723
Total	4904	1974	385	122	140	7525

† Excludes fires from Jan 1 to Mar 31, 2015 (n = 38). Size class is based on the final area burned measured after the fire is extinguished

Table 2 shows our variables, their descriptions, and sources. The majority of our variables are from Alberta Wildfire’s data management system, FIRES. Fire-level observations of operations and expenditure data were combined with geospatial data from public databases (Altalis, Government of Alberta GENESIS, Statistics Canada) to create a high-dimensional final dataset with detailed information on suppression costs, reporting delay, and the fire environment. Expenditure values that are originally reported in current year dollars have been set to 2020 dollars to account for inflation.

**Table 2:** Variables definitions and sources.

Variable	Definition	Source
Costs	Suppression expenditure per wildfire (2020 dollars)	FIRES
Reporting delay	Hours of delay between fire ignition and report	FIRES
Fire environment		
Weather	Weather variables are max/min/total on assessment day +/- 2 days	
Temperature	Maximum temperature (°C)	FIRES
Wind speed	Maximum wind speed (km/h)	FIRES
Rain	Total rainfall (mm)	FIRES
Humidity	Minimum relative humidity (%)	FIRES
Fuel type	DV* for *Timber* or *Slash* fuel types (baseline: *Open* (grass, peat, moss) or *Manmade*)	FIRES
Fire type	DV* for *Crown* fire type (baseline: *Ground* or *Surface*)	FIRES
High elevation	DV* for elevation over 1250 m	Altalis
South aspect	DV* for mean aspect between 157.5–202.5°	Altalis
Lake/River	DV* for lakes/rivers (within 3km). Surface water can serve as natural boundaries, and can be used to suppress fire.	GeoDiscover

Notes: * DV refers to dummy variables where 1 indicates the presence of the attribute; 0 for absence (or baseline). Data sources are as follows. FIRES: the Alberta Wildfire data management system. We received both publicly available and internal datasets for this project. (Publicly available data is available on www.alberta.ca/wildfire-maps-and-data.aspx). Altalis: a service that stores and publicly distributes the Government of Alberta’s comprehensive digital data sets. (www.altalis.com). GeoDiscover: Government of Alberta’s database for publicly available geospatial data (formerly GENESIS). Refer to https://geodiscover.alberta.ca/geoportal and open.alberta.ca/interact/geodiscover-alberta.

### 3.1 Suppression costs and reporting delays

For each fire, the data allow us to calculate the expenditures of: i. operating or renting aircraft and equipment, ii. salaries and wages for wildland fighters directly linked to each suppression mission, and iii. associated incidental costs (e.g. providing meals and accommodations for staff on extended deployment).

**Fig 3:**
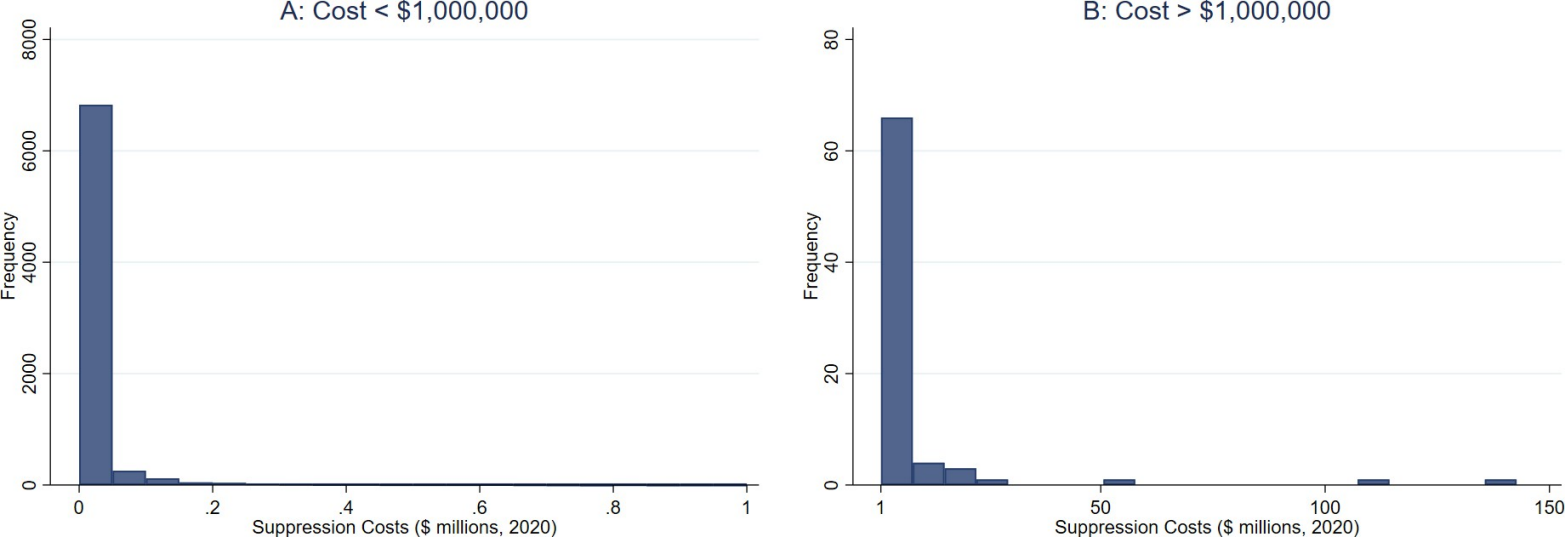
Wildfire suppression cost distribution.

Empirical modeling of wildfire suppression expenditures is challenging for several reasons. First, the distribution of costs is significantly skewed to the right. Fig 3 shows the cost distribution by splitting the data into two subsamples: less than and more than one million dollars. In both cases, the majority of the data is concentrated on lower cost levels, with a few extreme points.

**Fig 4:**
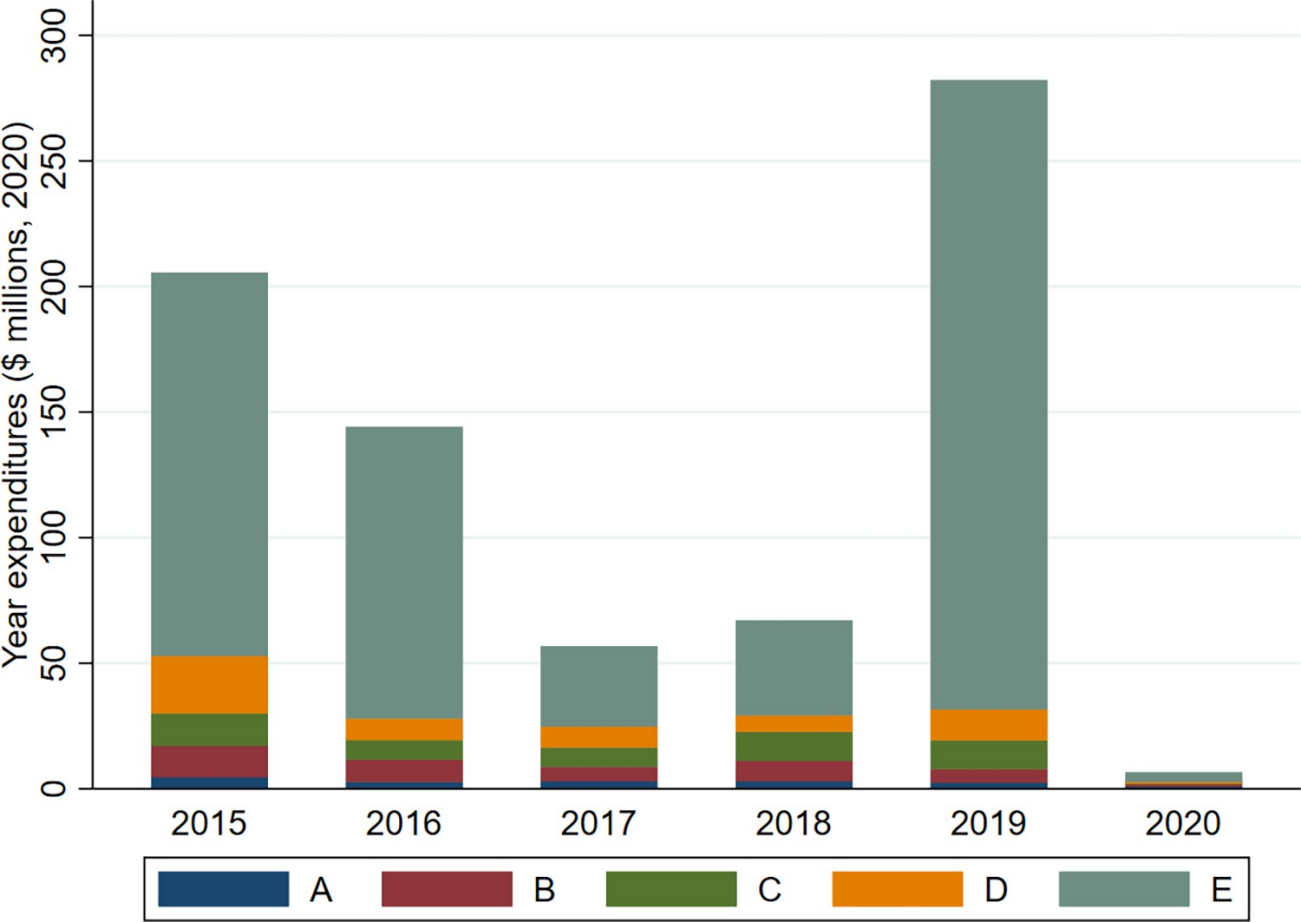
Yearly suppression operation expenditures, by size class (millions of 2020 dollars).

Even a single extreme wildfire, as is common in recent years, may dominate a sizeable share of annual expenditures, skew the data, and make it challenging to fit wildfire suppression expenditure models. Fig 4 shows the total suppression operation expenditures, by size class, from 2015–2020. As an example of outlier wildfire events, in 2019, the 21 extremely large fires (>200 ha) represent only 2.1% of total fire counts (see Table 1), yet exceeded 88% of total fire suppression cost. Moreover, while the total cost for suppression of all 2019 fires was $282 million, half of this amount is accounted by one individual wildfire.

Second, fire size is an endogenous variable in empirical models of suppression expenditures. While fire size is an important predictor of suppression expenditures, suppression effort (and consequently its costs) will influence fire behavior and ultimately impact the final area burned. This constitutes reverse causality and generates biases in the estimates of all coefficients in the model. That is, if the objective is to estimate the impact of reporting delay on suppression costs, controlling for fire size will introduce bias.

While we fully expect the omission of fire size from the cost model to reduce its predictive power, such a strategy removes endogeneity bias due to reverse causality. However, failing to control for fire size would still generate bias if the final area burned is affected by wildfire reporting delays, i.e. omitted variable bias. While this is a theoretical possibility, we do not find support in the data for such a hypothesis.

**Fig 5:**
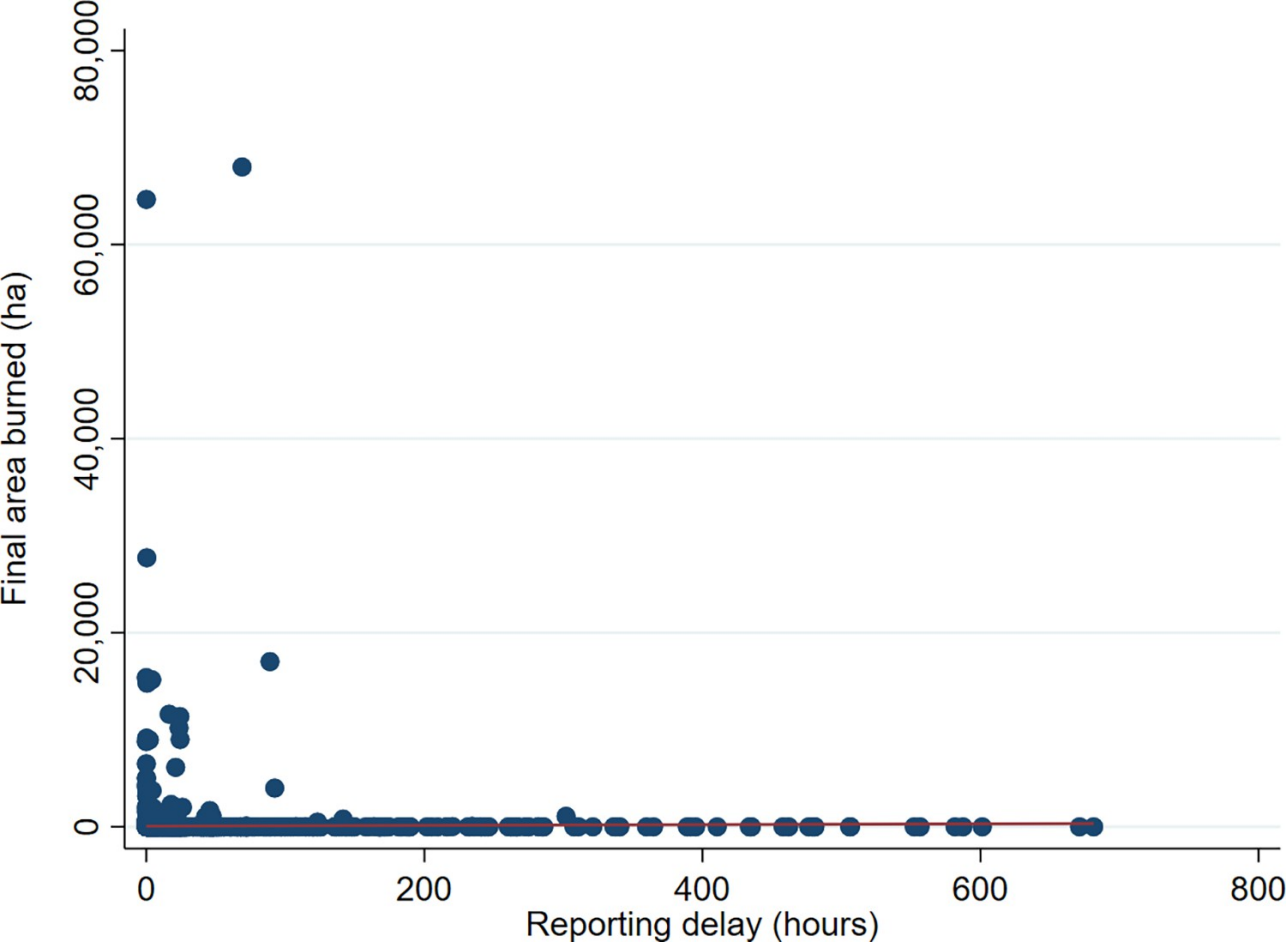
Scatter plot of final area burned and reporting delay.

We perform several analyses to investigate the nature of the relationship between fire size and reporting delay. Fig 5 shows the scatter plot of the final area burned and reporting delays (remark: to improve presentation, the figure omits three fires with final area burned greater than 200 thousand hectares). While the figure shows a wide range of reporting delays, we find no evidence that fire size increases with their reporting delays. This informal visual inspection suggests that the two variables are uncorrelated. A formal test is obtained by estimating a liner regression of fire size on reporting delay. We cannot reject the null hypothesis of no statistical relationship between the two variables (the slope coefficient is equal to 0.377, with p-value 0.402).

The analysis above examines the intensive margin of the relationship between fire size and reporting delay. We further investigate possible correlations between the two variables by examining the extensive margin. Specifically, we investigate the possibility that reporting delays play a role in determining fire size classifications. Because size class is a discrete and ordered variable, we use ordered probit models to test whether reporting delay influences the probability that a fire evolves to a higher size class. We estimate models with and without controls (weather, fuel type, topography–see Table 3). In both cases, we cannot reject the null hypothesis of no influence of reporting delay on fire size class (p-values are 0.179 and 0.240 for models with and without controls, respectively).

**Table 3:** Summary statistics, by fire size class.

	N	Mean	Std Dev	Min	Max
*Size class A (<0*.*1ha)*					
Cost (2020 Canadian dollars)	2965	4,945.45	7,760.77	6.78	127,382.41
Log cost	2965	7.24	1.92	1.91	11.75
Reporting delay (hr)	2965	12.61	51.87	0.00	681.36
Temperature (C)	2965	20.75	5.51	-9.10	33.00
Wind speed (km/h)	2965	16.79	6.22	3.50	50.00
Rain (mm)	2965	1.48	3.26	0.00	40.27
Relative Humidity (%)	2965	36.42	10.72	10.00	89.00
Fuel type: Timber slash	2965	0.56	0.50	0.00	1.00
Fire type: Crown fire	2965	0.02	0.13	0.00	1.00
South Aspect (true south)	2965	0.20	0.40	0.00	1.00
High elevation	2965	0.12	0.33	0.00	1.00
Lake/River within 3km	2965	0.33	0.47	0.00	1.00
*Size class B (0*.*1 to 4ha)*					
Cost (2020 Canadian dollars)	1645	22,667.05	33,033.79	26.32	311,946.00
Log cost	1645	8.94	1.80	3.27	12.65
Reporting delay (hr)	1645	10.01	38.53	0.00	478.74
Temperature (C)	1645	20.88	5.72	-2.50	33.40
Wind speed (km/h)	1645	17.33	6.43	0.00	47.00
Rain (mm)	1645	1.05	2.50	0.00	27.93
Relative Humidity (%)	1645	34.91	10.21	11.00	100.00
Fuel type: Timber slash	1645	0.61	0.49	0.00	1.00
Fire type: Crown fire	1645	0.06	0.23	0.00	1.00
South Aspect (true south)	1645	0.21	0.41	0.00	1.00
High elevation	1645	0.05	0.22	0.00	1.00
Lake/River within 3km	1645	0.31	0.46	0.00	1.00
*Size class C (4 to 40 ha)*					
Cost (2020 Canadian dollars)	324	148,458.20	174,134.82	21.89	976,246.13
Log cost	324	11.02	1.75	3.09	13.79
Reporting delay (hr)	324	10.49	35.75	0.00	336.31
Temperature (C)	324	22.27	5.84	0.70	31.70
Wind speed (km/h)	324	17.70	6.58	5.00	43.00
Rain (mm)	324	1.05	2.76	0.00	25.23
Relative Humidity (%)	324	33.97	9.62	12.31	59.67
Fuel type: Timber slash	324	0.72	0.45	0.00	1.00
Fire type: Crown fire	324	0.21	0.41	0.00	1.00
South Aspect (true south)	324	0.23	0.42	0.00	1.00
High elevation	324	0.07	0.25	0.00	1.00
Lake/River within 3km	324	0.19	0.39	0.00	1.00

Collectively, the results above suggest that reporting delay is not a significant determinant of fire size. This finding is largely in line with recent findings regarding the determinants of wildfire size in Alberta. Tymstra et al. (2021) analyze 80 large spring wildfires (>1,000 ha) across three decades and attribute dry, windy weather patterns as strong drivers of fire spread [37]. Using a sample of lightning-caused fires in Alberta, Tremblay et al (2018) apply survival analysis to quantify the effects of weather, fuels, and fire suppression activities on fire size [38]. They find that weather conditions such as low fuel moisture and high wind speeds are strongly associated with fire size. More recently (2023), Alberta faced an unprecedented fire season in which 36 fires over 10,000 ha contributed to a total of 2.2 million hectares area [39]. Beverly and Schroeder (2024) recognize that fire prevention and detection could be improved, however, they contend that the vast scale and range of fires in 2023 meant that suppression resources had to be triaged. Strategies to prioritize the protection of communities and other high-value assets led to a scenario in which many fires, which would have otherwise been addressed, were allowed to grow in size [39].

A final evidence regarding the fact that reporting delay is orthogonal to fire size class comes from testing for mean differences between reporting delays of fires from the different size class. We perform pairwise tests of the following null hypothesis: mean delay of class *i* minus mean delay of class *ii* equals to zero, where *i* and *ii* represent different size classes. None of the differences in means are statistically significant at the 5% level. This is evidence that fires that grow and eventually produce a large burned area are not detected with different times (on average).

In summary, the arguments above suggest that unspecified fire size is orthogonal to reporting delay in our suppression cost model. As such, we omit fire size to avoid reverse causality bias. Moreover, because Alberta Wildfire protocol specifies minimum levels of suppression resources for each fire class, we estimate the impact of reporting delays on costs by fire class. While Alberta Wildfire recorded over 7,500 fires from 2015 to 2020, we focus on fires of class A (<0.1 ha), B (0.1–4 ha) and C (4–40 ha), because, compared to size D and E fires, both the multitude of observations and the relative uniformity of expenditures make the subset of fires in class A-C more conducive to analysis by ML models.

**Fig 6:**
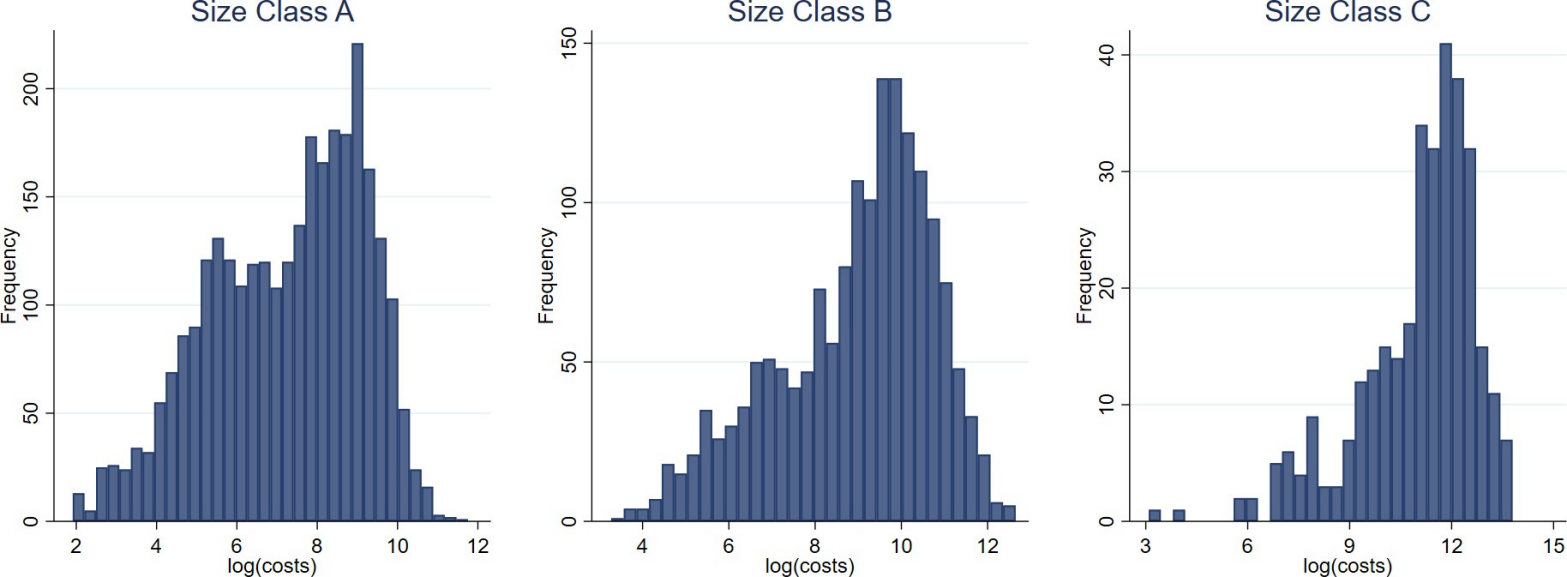
The distribution of log(costs), by size class.

Following previous wildfire expenditure research [40–44], log transformation is used both to make the cost distribution more symmetric and to address challenges related to heteroscedasticity. As well, the log-linear specification allows us to interpret the effect of reporting delay as a percentage of the suppression expenditure. Fig 6 shows the distribution of the logarithm of suppression costs, by fire size.

Finally, wildfires respond in complex and nonlinear ways to variation in the fire environment. We have no information on the shape of the influence of weather, fuel type, and the topography of the land area on suppression expenditures. As we discuss below, instead of making arbitrary parametric assumptions, our approach involves using Random Forests to nonparametric recover this relationship from the data.

### 3.2 Fire environment

Alberta Wildfire also provided a dataset with daily weather observations for 499 weather stations throughout the Forest Protection Area, across the timeframe of our study period. While some papers use a fire weather index to control for weather conditions [40, 43, 45], we follow Bayham and Yoder and calculate individual weather variables from raw information in the Alberta Wildfire weather dataset [46]. The weather variables are created using observations of every weather station within 50 km from the coordinates of the fire ignition point. Establishing a distance threshold is important because the range and accuracy of wildfire weather stations is highly dependent on local conditions, and after consulting Alberta Wildfire we were unable to determine range precision for stations in our dataset. At the 50 km buffer, stations cover 7,522 of 7,525 ignition points in our dataset. For a deeper discussion, the reader should refer to papers in the fire weather literature [47–49].

We recognize there is the possibility of two-way causal relationship between fire weather and suppression costs, particularly in large, long-lasting fires. In such scenarios, it is possible that weather conditions drive fire behaviour, which in turn affects suppression costs, while simultaneously, costs reflect suppression efforts that will alter fire growth and behaviour, which in turn can impact regional weather conditions. To mitigate endogeneity, we focus on weather observations that drive a fire in its initial period, which we define as five days centered on the assessment date. In summary, we calculate maximum temperature, maximum wind speed, total precipitation and minimum relative humidity.

We control for the influence of topography on fire behavior using calculated aspect and elevation variables [40]. These variables are sourced from Altalis, a public-private provider of geospatial data. Using the 100-metre raster projection of the Alberta Provincial Digital Elevation Model, we create a dummy variable equal to 1 for fires whose origins are on the south aspect of a slope (between 135–225°, where the landscape receives the most sun exposure), zero otherwise. We also create a dummy indicator for fires located in high elevation (over 1250 m, which is approximately the elevation of the start of the eastern slopes of the Rocky Mountains).

### 3.3 Summary statistics

As we focus on the impact of reporting delay on fires that received suppression expenditure, we exclude fires that had zero dollars for suppression (n = 839). Additional observations have been excluded when values were missing in their data source, such as fires for which there were no reliable weather data, as Alberta Wildfire stations were too far away (n = 274), and those for which fuel type were not recorded by Alberta Wildfire (n = 895).

Table 3 reports summary statistics for the fire-level variables, by fire size class. As expected, the average suppression costs increase with fire size. For class A fires, average cost is $4,945 (Canadian dollars of 2020). This value increases to $22,667 for class B, and to $148,458 for class C fires. The most expensive fire in our sample amounted to almost one million dollars in suppression costs. Average reporting delay hovers between 10–12 hours. A small proportion of fires are reported with no delay, i.e. 145 size A fires, 46 size B, and 10 size C. Both suppression costs and reporting delays have significant variance and their coefficient of variation is above 1 in all cases. The goal of the paper is to exploit this high variability to associate variation in suppression costs with variation in reporting delays, within fire size class, and holding constant fire environment, e.g. weather and landscape.

Regarding weather variables, average maximum temperature at fire ignition is about 21 degrees Celsius for fires type A and B, but about 1.5 degrees higher for fires that grow to class C. Wind speed, precipitation, and relative humidity are, on average, similar between size classes. Larger fires have more challenging fuel and fire type profile. Fifty six percent of fires in class A are fires that have timber slash as their primary fuel type. This statistic increases to 61% for class B and 72% for class C. Similarly, crown fires make up only 2% of class A fires, but 6% of class B fires and 21% of class C fires. Finally, regarding terrain topology, about 20% of fires start in locations with south aspect, and between 5–12% start in areas with high elevation. The presence of a water body near the location of a fire can be a significant factor in explaining suppression costs. While about 31–33% of fires size class A and B have a lake or river nearby, for size class C, only 19% of fires are within 3 km from bodies of water.

The next section discusses the machine learning method we use to estimate the impact of fire detection delays on fire expenditures.

## 4. Methods

The goal of the paper is to estimate the impact of reporting delays on suppression costs, holding the fire environment constant. We are interested in the following system,

$$Y_{it}=\beta D_{it}+g(X_{it},\delta_{t})+\varepsilon_{it}$$ (1)

$$D_{it}=m(X_{it},\delta_{t})+\mu_{it},$$ (2)

where Eq (1) is the outcome equation and Eq (2) models wildfire detection. Specifically, *Y*_*it*_ represent (log of) suppression cost of fire *i* in season *t*,*D* is reporting delay, *X* are confounding variables that describe the fire environment, *δ* captures season effects, *ε* and *μ* are zero-mean error terms. The function *g*(.) captures the influence of the fire environment and fire season on suppression costs. Importantly, Eq (2) allows for the fire environment and the fire season to influence reporting delays via the function *m*(.).

While the system above is conceptually flexible, it creates empirical challenges due to the nature of the relationships between fire environment, suppression costs, and reporting delays, i.e. the nuisance parameters *g*(.) and *m*(.) may represent complex and highly nonlinear relationships. For example, the econometrician does not know the true functional form of the influence of temperature, wind speed, elevation, etc. on the suppression outcome, nor how these variables may influence reporting delays. While our focus is on the impact of reporting delays on costs, which is measure by *β*, misspecification of nuisance parameters can bias the relevant policy estimates.

To avoid parametric misspecification bias, empirical approaches should rely on nonparametric estimation of *g*(.) and *m*(.). Kernel regressions are a classic nonparametric method to perform such an estimation. However, slow convergence rates due to the curse of dimensionality make these nonparametric regressions less appealing in small sample applications such as ours [50]. Machine Learning (ML) offers state-of-the-art nonparametric approaches to fit the functions *g*(.) and *m*(.) to the data. In this paper, we use Random Forests to fit the functions *g*(.) and *m*(.) [51].

Tree-based models are among the most applied ML techniques [52]. Random Forests employ multiple decision-trees on many sub-samples of the data, with random subsets of the features for node splits. In regression problems, the method uses averaging to improve predictive accuracy [51]. As Random Forests ensemble multiple decision trees, they tend to reduce overfitting and more adequately fit nonlinear relationships [53]. Random Forests have been instrumental in empirical work in a wide array of wildfire applications. A review of the ML literature found that Random Forests are the most used algorithm in wildfire science [54]. For example, many papers have used Random Forests to perform fuel classification [55–57] and lightning prediction [58]. Random Forest models have also been shown to have superior performance in burned area assessment [59].

However, the direct application of a ML algorithm in the context of our system is challenging. The issue is related to the estimation of a one-dimensional causal parameter *β* in a model where high-dimensionality parameters such as *g*(.) and *m*(.) are unknown and must also be uncovered from the data using ML. Chernozhukov and co-authors show that regular ML can be applied to the system above and successfully predict *Y* and *D*, however, the estimate of the one-dimensional parameter is severely biased [28]. This happens because ML implements regularized estimators in order to optimize prediction and off-the-shelf algorithms are not designed to obtain a causal (unbiased or consistent) estimate of a single parameter. In our application, this parameter is the main parameter of interest, *β*: the impact of reporting delay on suppression costs.

Chernozhukov et al. propose the implementation of Double Machine Learning (DML) to de-bias the estimate of *β*. Based on a Frisch-Waugh-Lovell approach, the DML addresses regularization bias using orthogonalization. The procedure involves three steps: i. use Random Forests to predict *Y* from *X* and *δ* (fire season dummies); ii. similarly, obtain Random Forests predictions of *D* from *X* and *δ*, and finally; iii. perform a residual-on-residual regression to obtain a bias-free estimate of *β*. The DML also involves sample splitting and cross fitting to reduce overfitting bias and addresses issues of loss of statistical power. In this paper, we adopt the following orthogonalization algorithm to estimate *β*.

- Randomly split the sample into two subsamples, namely A and B.
- Using subsample A:
  - compute the Random Forest prediction ${\hat{Y}}^{A}$ from *X*^*A*^ and *δ*^*A*^
  - compute the Random Forest prediction ${\hat{D}}^{A}$ from *X*^*A*^ and *δ*^*A*^
- Using observations *Y* and *D* from subsample B:
  - compute residuals $y_{B}^{A}=Y^{B}-{\hat{Y}}^{A}$
  - compute residuals $d_{B}^{A}=D^{B}-{\hat{D}}^{A}$
- Estimate ${\hat{\beta }}_{B}^{A}$ *a la* Frisch-Waugh-Lovell by linear regression of $y_{B}^{A}$ on $d_{B}^{A}$, where ${\hat{\beta }}_{B}^{A}$ is the slope coefficient
- Repeat steps II-IV by reverting the sample roles to compute ${\hat{\beta }}_{A}^{B}$
- Compute ${{\hat{\beta }}_{j}=(\hat{\beta }}_{B}^{A}+{\hat{\beta }}_{A}^{B})/2$
- Repeat steps I-VI *J* times (we use *J* = 400)
- Finally, compute $\hat{\beta }=\sum_{j}\frac{{\hat{\beta }}_{j}}{J}$.

The Random Forest predictions in step II are obtained using the R package Generalized Random Forests–GRF [60]. The package uses cross-validation to tune the following training parameters:

- Tree-growing parameters:
  ○ the fraction of the data used to grow trees (the number of trees is set to 2000)
  ○ the number of variables in each split
  ○ the minimum number of observations in each leaf
- Honest splitting [61]:
  ○ whether to implement sub-sample splitting, also known as ‘honest forests’, i.e. an additional split into halves of the fractional subsample, one for tree splitting (constructing the trees) and one for populating the leaf nodes (making predictions)
  ○ The fraction of the honest split used to select tree splits is also tuned within the range [0.5–0.8]
  ○ whether or not to prune away empty leaves after training
- Split balance parameters:
  ○ the maximum imbalance of a split (*α*), i.e. the size of a child node must be at least α*size(parent)
  ○ an imbalance penalty (*δ*) to discourage child nodes from having very different sizes; *δ* *(1/ size(left.child) + 1/ size(right.child))

The cross-validation procedure uses one hundred random draws in the parameter space, trains forest for each set of parameters, and computes out-of-bag errors. Next, a smoothing function of errors is estimated and the values that minimize smoothed errors are chosen as the parameters of the tuned model (refer to https://grf-labs.github.io/grf/REFERENCE.html for additional details on parameter tuning).

The approach above produces an empirical distribution of the DML parameter *β* that can be used for statistical inference and hypothesis testing. To control for size effects and unobserved heterogeneity in environmental and policy factors due to size class, we apply our algorithm separately across subsets of size class A, B, and C.

## 5. Results

**Fig 7:**
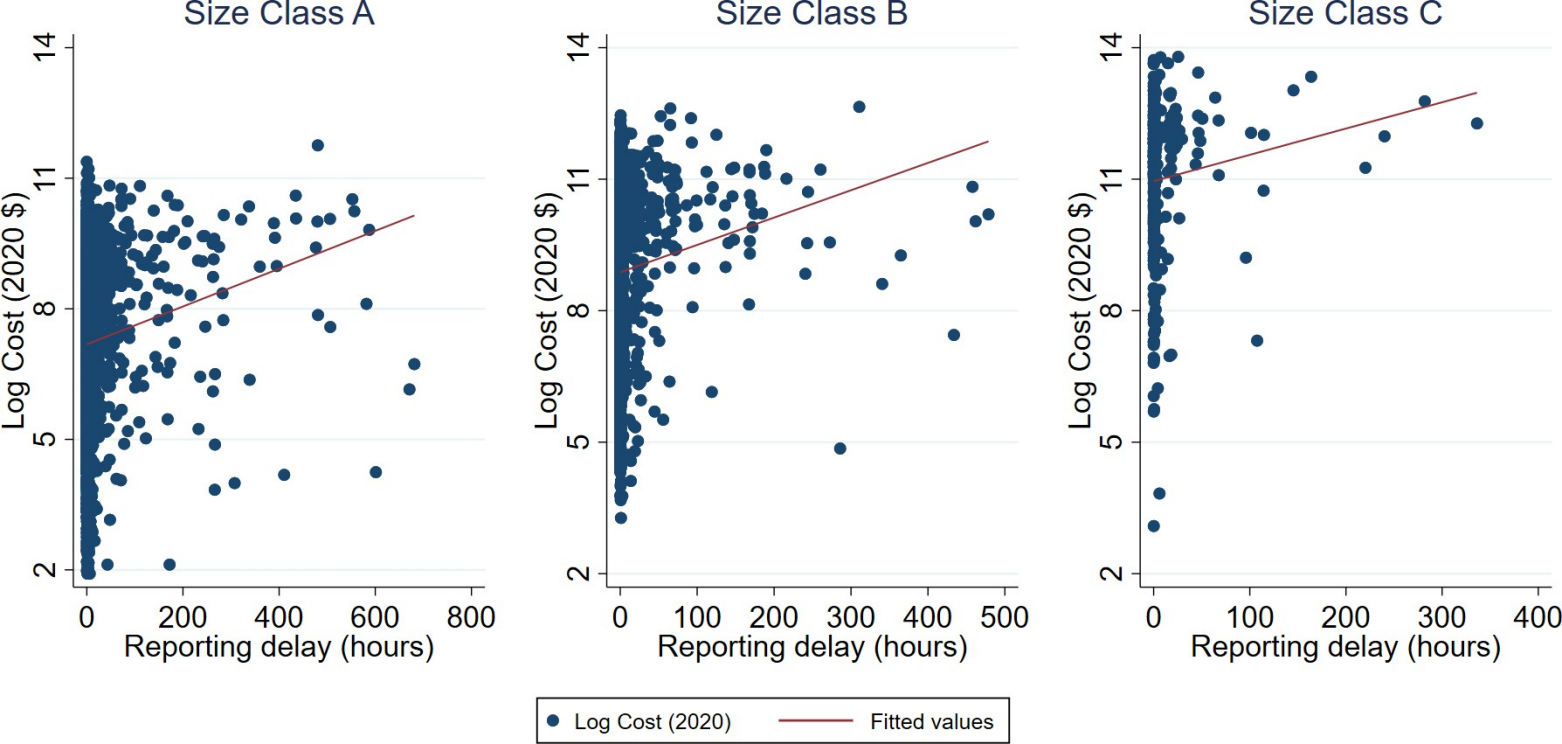
Log cost and reporting delay, by fire size class.

Fig 7 shows scatter plots of log costs on reporting delay, by fire size class. Observations in which reporting delay exceeds 30 days have been excluded due to concerns with transcription errors in the dataset as well as to eliminate outliers. The figure shows the linear prediction of costs from reporting delays. This preliminary analysis reveals a positive correlation between the two variables, i.e. larger reporting delays are associated with larger suppression costs.

Table 4 reports the estimates $\hat{\beta }$ of the impact of reporting delay on the log of suppression cost, by fire size class. The results indicate that one additional hour of reporting delay increases suppression costs by 0.256% for fires of size class A (p<0.01), 0.246% for size class B (p<0.01), and 0.280% for size class C (p<0.01).

**Table 4:** Estimates of the impact of reporting delay on ln(suppression costs), by size class.

	A	B	C
$\hat{\beta }$	0.00257***	0.00244***	0.00270***
	(5.965648e-06)	(1.007552e-05)	(3.364487e-05)
N	2,965	1,645	324

*Notes*: Fire size classes: A: <0.1 ha; B: 0.1–4 ha; C: 4–40 ha. Standard errors in parentheses.

* p<0.10

** p<0.05

*** p<0.01.

While the coefficients are similar in magnitude across size classes, their economic significance varies. For example, given that the average expenditure incurred in the suppression of a size class A fire is $4,945.45, an additional hour of delay increases total suppression cost by $12.66. For size class B, the average cost is $22,667.05, so the additional cost of an hour of delay is $55.76. Finally, for size class C with average cost of $148,458.20, the marginal cost increment is $415.68.

The numbers above can also be used to predict, on average, the contribution of wildfire detection to total suppression costs. Class A fires are detected with an average reporting delay of 12.61 hours. Using the marginal (or per hour) valuation estimate of $12.66, the contribution of reporting delays to suppression costs is, on average, only $159.64 per fire, which represents only 3.2% of the average suppression costs. Using the same method, the contribution of reporting delays to the costs of suppression of fires in size class B is, on average, $558.19 per fire, or 2.5% of average costs. Finally, for size class C, reporting delays cost $4,360.48 per fire (on average), which represents 2.9% of the average total suppression costs.

## 6. Discussion

While early detection is generally assumed to be a critical component in cost-effective wildfire suppression (refer to Duff et al. (2015) [62] for a review), empirical literature on the effect of early detection on suppression cost is limited [62–64]. In performing a cost-benefit analysis of lookout towers in Wisconsin, Steele and Stier find that while the cost of operating lookout towers exceed their value in the reduction of direct suppression cost, the benefit of towers are realized in the reduction of total wildfire costs, which also include property loss [64].

Since the work of Steele and Stier, only a few studies have incorporated reporting delay (measured in time units) into a suppresion expenditure empirical model [40–42]. This literature reports mixed results with the effect of detection delay on costs typically not significant. As a part of a PhD dissertation, Lankoande applies linear regressions to a dataset of consisting of over 307 thousand fires from 1970 to 2002 covering the continental United States [42]. Three models are created in which suppression costs, burn area and damage costs are modeled on detection delay time, along with environmental, weather and population density variables. Detection delay time is expressed in units of log and log of the squared delay variable. The results indicate the puzzling effect that an increase in detection delay significantly decreases both suppression costs and the burn area at an increasing rate, but increases damage costs at a decreasing rate.

Acknowledging the model specification in Lankoande, Gebert et al. apply a further detailed linear regression model to a highly multi-dimensional dataset of US Forest Service-suppressed wildfires from 1995 to 2004 [40]. The dataset includes 1,550 fires that had escaped initial containment, from 100 acres (40 ha) to over 300 acres (121 ha). The authors examine suppression expenditure per acre as the dependent variable, stating that fire managers were accustomed to considering cost as per-unit area measurement. After separating the dataset into West/East regions, Gebert et al. model the suppression cost per acre for each wildfire as a function of many variables including the fire environment, values at risk, resource availability and reporting delay. Delay is included in both in log and squared log form. The delay effect was not significant in West, however in the East, a longer delay period decreases suppression cost per area up to 22.6 hours, at which point delay increases cost per area.

More recently in 2022, MacMillan et al. apply both parametric and nonparametric methods for suppression expenditures of 5,459 fires in the Canadian province of British Columbia, which neighbours Alberta, from 1981 to 2014 [41]. The study uses a negative binomial regression to address the non-normal cost distribution, right-skewed by extremely expensive fires. Nonparametric ML models include Random Forests as well as gradient boosting. When both parametric and nonparametric models are used to forecast out-of-sample fires, the researchers find that the models have similar predictive performance. Results from both parametric and ML models indicate that the effect of log detection time delay is marginally significant (p<0.10) in slightly reducing expenditure. MacMillan et al propose that the negative effect of delay on cost may reflect the possibility that fires in remote areas receive lower fire management priority.

Our analysis of the detection-expenditure nexus excludes large wildfires. As discussed above, the relationship between suppression expenditures and wildfire size is largely nonlinear (see Fig 4). Typically, the development in size (and consequentially suppression expenditure) of large-scale wildfires are attributed to factors beyond the reporting delay during the initial phases of a response protocol. This observation is substantiated by the findings of an external review commissioned by Alberta Wildfire for the notable 2016 Horse River (Fort McMurray) Wildfire (size class E at over 600 thousand hectares). The review determined that fire development was due to a combination of environmental factors (low relative humidity, high temperatures, high wind speed and gusts), as well as concurrent fires in the region that also required suppression resources. Further, the report concludes that “time lag is not believed to be inordinately long as the wildfire was detected at a size of less than 2.0 hectares–within expectations set for the detection program” [65]. Given the potential for a myriad of new variables to arise with the size of a fire complex, especially for fires that significantly deviate from seasonal norms, in this study, we focus on an analytical sample of 4,934 fires ranging from size class A to class C. By narrowing our scope, we discover the specific impact of reporting delay on suppression costs, for fires that have been successfully suppressed before reaching a critical size.

## 7. Conclusion

The Double/Debiased Machine Learning (DML) approach delivers precise estimates of the policy parameter *β*. The results indicate that reducing the delay between ignition and reporting of a wildfire delivers statistically significant (albeit modest) effects in reducing suppression expenditures; $\hat{\beta }\approx 0.0026$ (p<0.01). Contrary to the previous studies that show reporting delay to have a negative effect on suppression costs [40–42], in our DML model we find that a reduction in reporting delay decreases fire suppression expenditure. Specifically, we find that each hour of reporting delay reduction tends to reduce the cost of suppression by approximately 0.25% (p<0.01).

These results give decision makers in Alberta Wildfire empirical evidence that investments in improving early detection has discernable payoffs in reducing total suppression cost. For instance, in our dataset, the average reporting delay of a size C fire in 2019 was 6.77 hours. If reporting delay was reduced by one hour for each of the 54 size C fire observations in this subset, total expenditure across all fires could have been reduced by $31,000. This reduction represents a modest proportion of suppression costs, as the average cost for suppressing a size class C fire in 2019 was $201,800.

Nonetheless, it is possible that the benefits of early detection and reporting are further realized when taking into account the reduction of total wildfire damage costs, which includes loss of property and assets [64]. Taking into account our empirical results on suppression expenditure savings, as well as the consideration of further possible reductions in wildfire damage costs, wildfire managers are better informed to consider how worthwhile it may be to upkeep existing detection systems (i.e. lookout towers and reporting lines), and the possible utility of adopting new tools like UAS and other automated technologies.

In recent years, new technologies like UAS and deep-learning are showing promising steps to reducing the impact of wildfires [66–68]. Whatever are the tools being utilized, our research suggests that initiatives to reduce reporting delays have statistically significant impacts (albeit modest in magnitude) on reducing the costs incurred in fire suppressions. As such, investments to improve the detection of wildfires and reduce reporting delays are more reasonably justified on the basis of socioeconomic gains beyond the savings that prompt detection generates on suppression costs.

### 7.1 Limitations

We recognize that economic modelling of suppression costs continues to have its limitations, despite the methodological improvements of new frameworks like DML. Wildfire suppression is a complex topic, and the interaction between environmental and human variables are challenging to model. For instance, effects such as media coverage or political influence have been proven to impact suppression costs [69], though such effects are not addressed in our models. In our study, we have excluded fires that are difficult to account, such as those formed as part of a larger “fire complex”. However, we must recognize that it is often extreme fire events that drive the bulk of wildfire expenditure, highlighting the need for further examination of fire complexes.

While the paper investigates the potential to influence suppression costs via early wildfire detection using a machine learning model that controls for nonlinear effects of the fire environment, our approach is not able to incorporate data of larger fires (class D and E). This is a significant limitation as those fires are responsible for a disproportionate share of suppression expenditures. Nevertheless, our findings suggest that suggests that reporting delay is not causing fires to grow to a higher fire class. Future work is need to address the challenges of working with large fire data, including the skewedness of the cost distribution and the fact that certain elements of the fire attack may be responsible for the bulk of expenses (e.g. aircrafts) thus reducing the variation available to estimate the impact of other observables.

The cost of operating air tankers, helicopters, and other aircraft make up a substantial portion of suppression expenditures of large wildfires; aircraft is critical both in directly tackling flames and in limiting the spread of fire via laying retardant barriers retardant [70, 71]. Future work is necessary to investigate viable alternatives to the deployment of aircraft [72], and to define the metrics for assessing the supply and demand of suppression resources [73].

Our approach to estimate impacts by fire size class, while beneficial for accommodating the skewedness of the data, suffers from limitations. Since we do not model what determines the size of each fire, our models are estimated in selective samples. As such, our estimates do not represent the impact of reporting delay on the cost of a random fire. Instead, the paper provides an impact estimate for each type of fire. While conceptually less general, in practice our approach is informative since we find similar results across the different types of fires, i.e. our impact estimates are equal to 0.0026, 0.0024, and 0.0027 for fires size class A, B, and C respectively. In other words, for the fires in our sample, an increase in detection delay of one hour leads to an increase in suppression costs of about 0.025%.

Finally, another limitation of the model is the linearity assumption of in Eq (1). While the outcome equation is flexible regarding the influence of fire environment on suppression costs, the model adopts a parsimonious approach regarding the influence of reporting delays on costs. While the simple linear parametrization makes β estimates easy to interpret, it is possible that, even after controlling for nonlinear effects of fire environment, the effects of reporting delays on costs can be nonlinear. Future work is needed to adapt the methods employed in this paper to accommodate this additional source of nonlinearity.
